# *Pseudomonas aeruginosa*: A typical biofilm forming pathogen and an emerging but underestimated pathogen in food processing

**DOI:** 10.3389/fmicb.2022.1114199

**Published:** 2023-01-25

**Authors:** Xuejie Li, Nixuan Gu, Teng Yi Huang, Feifeng Zhong, Gongyong Peng

**Affiliations:** ^1^School of Food Science and Engineering, Guangdong Province Key Laboratory for Green Processing of Natural Products and Product Safety, Engineering Research Center of Starch and Vegetable Protein Processing Ministry of Education, South China University of Technology, Guangzhou, China; ^2^Research Institute for Food Nutrition and Human Health, Guangzhou, China; ^3^Department of Diagnostics, Second Affiliated Hospital of Shantou University Medical College, Shantou, China; ^4^State Key Laboratory of Respiratory Diseases, National Clinical Research Center for Respiratory Diseases, National Center for Respiratory Medicine, Guangzhou Institute of Respiratory Health, the First Affiliated Hospital of Guangzhou Medical University, Guangzhou, China

**Keywords:** *Pseudomonas aeruginosa*, foodborne, pathogen, biofilm, antimicrobial resistance

## Abstract

*Pseudomonas aeruginosa* (*P. aeruginosa*) is a notorious gram-negative pathogenic microorganism, because of several virulence factors, biofilm forming capability, as well as antimicrobial resistance. In addition, the appearance of antibiotic-resistant strains resulting from the misuse and overuse of antibiotics increases morbidity and mortality in immunocompromised patients. However, it has been underestimated as a foodborne pathogen in various food groups for instance water, milk, meat, fruits, and vegetables. Chemical preservatives that are commonly used to suppress the growth of food source microorganisms can cause problems with food safety. For these reasons, finding effective, healthy safer, and natural alternative antimicrobial agents used in food processing is extremely important. In this review, our ultimate goal is to cover recent advances in food safety related to *P. aeruginosa* including antimicrobial resistance, major virulence factors, and prevention measures. It is worth noting that food spoilage caused by *P. aeruginosa* should arouse wide concerns of consumers and food supervision department.

## 1. Introduction of *Pseudomonas aeruginosa*

*Pseudomonas aeruginosa* is an aerobic, motile, slightly curved Gram-negative bacterium that is ubiquitous in diverse environments, including air, water, soil, and plant and animal tissues. As a well-known opportunistic human pathogen, *P. aeruginosa* possesses the ability to cause a whole host of severe acute and chronic life-threatening infections such as meningitis, otitis media, urinary tract infections, and pneumonia, especially in cystic fibrosis individuals. It is in the top three causes of opportunistic infections in humans and annually affects over 2 million patients and kills about 90 thousand each year (Cross et al., 1983). Because of the extracellular enzyme secretion capacity, *P. aeruginosa* is also a common spoilage bacterium, particularly in higher water content and nutrient-rich foods.

## 2. *Pseudomonas aeruginosa* is a leading human pathogen and super-bug

*Pseudomonas aeruginosa*, a ubiquitous opportunistic pathogen, could cause serious acute and chronic infections in immunodeficient patients, commonly in the bloodstream, urinary tract, respiratory tract, soft tissue, or wound. The pathogenicity of *P. aeruginosa* stems from diverse virulence factors and remarkable genetic flexibility which allows it to adapt to various habitats and escape host immune defenses (Klockgether and Tümmler, 2017). The emergence of carbapenem-resistant strains of *P. aeruginosa* is a sanitary health threat (Wang L. et al., 2021; Wang L. J. et al., 2021; Liu et al., 2022c) and a source of extreme concern to the World Health Organization (WHO). In 2007, the number of infections arising from *P. aeruginosa*, which is multidrug- or carbapenem-resistant, was approximately 23,575 and the number of deaths was approximately 1,573, according to reports from the EU region. And then in 2015, the number of infections increased to over 68,278, and the number of deaths increased to about 4,564 in these areas (Cassini et al., 2019). Therefore, the WHO considers study and exploitation of novel treatments for carbapenem-resistant *P. aeruginosa* to be of great urgency (Tacconelli and Magrini, 2021).

Bacterial virulence factors include lipopolysaccharide (LPS), adhesins, lectins, flagella, and so on, which contribute to the *P. aeruginosa* pathogenesis and disrupt signal transduction pathways in host cells. As a physical barrier, LPS causes tissue damage and antibiotic resistance due to its endotoxic activity (Chadha et al., 2022). The polar flagellum responsible for motility in liquid or on solid surfaces is also an important virulence factor. The attachment ability of the flagellum aids initial binding to the cystic fibrosis epithelia and initiates biofilm establishment (Jurado-Martin et al., 2021). The type IV pili play an essential role for *P. aeruginosa* in adhesion to several cell types, initiation of biofilm formation, and attachment to specific tissues (Barken et al., 2008). Pili is able to control twitching motility which is used for colonization on different cell surfaces. Among the five secretory systems secreting various hydrolytic enzymes and toxins to attack the host, the most important secretion system is type III, which directly injects the virulence factors into host cells and can also destruct the host’s immune system. Four well-known anti-host factors, ExoS (exoenzyme S), ExoT, ExoU, and ExoY are injected *via* the type III secretion system. ExoS mainly inhibits the function of neutrophils and type I pneumocytes in the early and later infection phase, respectively. 92%–100% of clinical isolates produce ExoT which impedes wound healing and inhibit cell division. ExoU, a phospholipase, can rapidly destroy the membrane of host cells and consequently leads to severe lung injury, proinflammatory response, sepsis, and mortality. ExoY irreversibly causes proapoptotic processes and actin microtubule disassembly (Jurado-Martin et al., 2021). Exotoxin A (ETA), an ADP-ribosyl transferase is the most toxic virulence factor of *P. aeruginosa* causing necrotizing at the site of colonization and inhibiting host cell protein synthesis. The various extracellular proteolytic enzymes and lipolytic enzymes all secreted by the type II secretion system are also weapons for *P. aeruginosa* invasion. Elastase A (LasA), a serine protease secreted by *P. aeruginosa* is proven to be relevant to antibiotic resistance in clinical isolates. The most abundant protease elastase B (LasB) is the major virulence factor. Due to its protein cleavage activity, LasB could interfere with bacterial clearance, disrupt epithelial junctions, and affect biofilm formation (Behzadi et al., 2021). To adapt to the diverse environmental conditions, lipases synergy with other virulence factors is expressed. *P. aeruginosa* has also been considered to be a “Super-bug,” as a number of antimicrobial resistance determinants and mechanisms have been commonly reported, including formation of biofilm, carriage of plasmids and integrons (Xu et al., 2021c).

## 3. *Pseudomonas aeruginosa* is a typical biofilm former

*Pseudomonas aeruginosa* is a critical biofilm-forming species and is also a paradigm bacterium for the investigation of biofilms. It was reported that 65%–80% of nosocomial infections were related to biofilms. Biofilm is a structure built primarily of autogenous extracellular polymeric substances (EPS) that serves as a scaffold to wrap bacteria on a surface and shield them from external pressures and hinder phagocytosis (Ma et al., 2022). Bacteria within biofilms demonstrate distinct properties from those of planktonic growth. Particularly, bacteria within biofilms are much less susceptible to antibiotics, disinfectants, and host defenses (Xu et al., 2021a). The EPS of *P. aeruginosa* biofilms matrix attached to a variety of surfaces or tissues consists of proteins, exopolysaccharides (Psl, Pel, and alginate), and extracellular DNA (eDNA) which can be hydrolyzed by DNase I (Swartjes et al., 2013). The process of biofilm formation by *P. aeruginosa* progresses begins with attachment to the surfaces suitable for growth including medical instruments and food, followed by the formation of microcolonies, and finally, maturation involving the expression of matrix polymers (Sauer et al., 2002). In addition, *P. aeruginosa* has been often found in polymicrobial interaction, including *S. aureus* in bacteria and *Candida albicans* in fungi (Liu J. et al., 2021).

## 4. *Pseudomonas aeruginosa* often exists in various food types

Despite being widely known, *P. aeruginosa* is a leading human opportunistic pathogen, in food safety areas it is an underrecognized microorganism. Due to its high metabolic versatility, rapid reproducible ability, high adaptive capacity, and growth abilities at low temperatures, *P. aeruginosa* is common worldwide and consequently is a prevalent causative agent of food infection (Gao et al., 2023).

### 4.1. Water

*Pseudomonas aeruginosa* usually exists in humid environments and is an important source of pollution in drinking water. In China, the presence of *P. aeruginosa* in a 250 mL sample of potable water is explicitly prohibited. At the same time, the United States, Europe, Japan, Canada, Brazil and the World Health Organization limit the amount of *P. aeruginosa* in potable water to the maximum possible number is less than 3 CFU/L or no *P. aeruginosa* can be detected in 250 mL water samples. The pollution of barreled potable water by *P. aeruginosa* is frequently reported and is gradually becoming the main indicator of unqualified barreled drinking water quality. Due to incomplete disinfection or cross-contamination of source water, water injection tanks, pipelines, filtration in the water production process, reverse osmosis facilities, etc., the recovery of empty barrels, the inadequate cleaning of the empty barrels, and the poor sealing of the lid are all possible causes *P. aeruginosa* to pollute the bottled water. The pollution of potable water by *P. aeruginosa* occurs from time to time, and symptoms such as vomiting and diarrhea may occur when drinking water contaminated by *P. aeruginosa*. *Pseudomonas aeruginosa* was identified in 3% of drinking water (Allen and Geldreich, 1975), 18.8% of bottled water, 9% of tap water, and 90% of sewage water samples (Raposo et al., 2016). In addition, 206 water samples collected from Guangzhou, China were tested for *P. aeruginosa* and the results showed 10 for finished water, 52 positive for source water, and 67 for carbonated water (Wu et al., 2016). In Paris, by testing water samples in 4 monomer distribution systems, *P. aeruginosa* was positive in 52.17% of the samples. Transformation, *P. aeruginosa* changes, and the isolation rate is relatively high from March to May (Perrin et al., 2019). In Venezuela, the isolation rate of *P. aeruginosa* is as high as 92.5% in drinking water (Table 1).

**Table 1:** Prevalence of *Psuedomonas aeruginosa* in different types of water.

Water type	Location	No. of tested samples	No. of positive samples	Infection rate	Author
Drinking water	Colorado			3%	Allen and Geldreich (1975)
	Guangdong, China	206	129	62.62%	Wu et al. (2016)
	Paris	368	192	12.20%	Perrin et al. (2019)
	French and German long-term care facilities	35	1	41%	Martak et al. (2022)
	Cape Town, South Africa	709	94	14%	Opperman et al. (2022)
Tap water	Hospitals in Northern Ireland	494	14	12.20%	Walker et al. (2014)
Sinks water	French and German long-term care facilities	254	31	2.90%	Martak et al. (2022)
Water				18.8% of bottled water 9% of tap water 90% of sewage water	Raposo et al. (2016)

### 4.2. Milk

The abundant nutrients such as protein, fat, carbohydrates, and vitamins make milk susceptible to contamination by a number of microorganisms. Due to the biofilm production ability facilitated it attaches to the wall of milk cooling tanks, milk cans, and pipes for milk delivery, *P. aeruginosa* is a bacterium that is often separated from milk. Consequently, milk is an efficient route for transmitting pathogens (Chen et al., 2011). Psychrophilic bacterium *P. aeruginosa* is able to continuously develop its population at the temperature of 4–6°C. The short generation time of less than 4 h at temperature of the 4°C of *P. aeruginosa* has enabled its number to increase to over 10^6^ CFU/mL in milk after 8 days, although contamination with only a single *P. aeruginosa* (Meesilp and Mesil, 2019). In Ethiopia, 54 different bacterial species were identified from 107 raw milk and pasteurized milk samples. Specifically, *P. aeruginosa* (18.5%), *Escherichia coli* (29.6%), and *Klebsiella pneumoniae* (16.7%) were detected in milk samples (Garedew et al., 2012). In the Czech Republic, 18 (8.87%) of *P. aeruginosa* were identified out of 203 samples of fresh milk and two strains (4%) were identified from 50 samples of pasteurized milk during a year. Exposed to pasteurization temperatures (72°C) for 20 s, *P. aeruginosa* would be devitalized. At refrigerator temperatures, strain cells multiplied by an average of two orders of magnitude (Mickova et al., 1989). *Pseudomonas aeruginosa* not only exists in cow’s milk but can also be detected in goat milk and camel milk. In pasteurized milk, the isolation rate of *P. aeruginosa* may also be relatively high, sometimes as high as 40% (Mickova et al., 1989).

### 4.3. Meat

Due to the abilities of proteolytic, lipolytic, and saccharolytic processes, *P. aeruginosa* dominates and spoils the meat. Fresh meat sold in the market is more likely to be contaminated by *P. aeruginosa*, which may be related to exposure, handling processes, and cleaning processes using contaminated water (Alimi et al., 2022). One hundred and five retail meat product samples, including fresh minced meat (*n* = 45), fresh sausage (*n* = 30), and frozen beef burger samples (*n* = 30) were collected from Cairo and Giza markets to isolate and identify *P. aeruginosa*. The examined meat product samples showed that *Pseudomonas* species were detected in 32 (71.11%), 13 (43.33%) and, 8 (26.67%) with an average count/g of 3.2 × 10^4^, 1.07 × 10^3^, and 2.2 × 10^3^ of the investigated samples, respectively, (Amal et al., 2014). From November to February, all 150 retail meat samples including beef (*n* = 50), mutton (*n* = 50), and chicken meat (*n* = 50) were collected in Mosul city to isolate *Pseudomonas* species. And the result showed that 53 *Pseudomonas* strains were isolates from all these meat samples examined (35.33%), including 46% (23) from beef, 22% (11) from lamb, and 38% (19) from chicken (Dong et al., 2022), with an average count/g of 1.47 × 10^4^, 1.92 × 10^4^, 2.13 × 10^5^ cfu/g, respectively (Tahr and Hasan, 2022). The occurrence frequency of *P. aeruginosa* is 3% from meat and meat product samples (*n* = 100) in north-central Nigeria (Alimi et al., 2022). In addition, camel meat may be contaminated by *P. aeruginosa*. In camel meat, the totality of *Pseudomonas* spp. was isolated at a rate of 10/100, 8/10 for *P. aeruginosa*, and 2/10 for *Pseudomonas fluorescens*, respectively. The isolation of *P. aeruginosa* in meat was not caused by camel infection with *P. aeruginosa*, because *P. aeruginosa* tested positive in 45 out of 200 healthy camel meat samples (22.5%; Elhariri et al., 2017; Table 2).

**Table 2:** Prevalence of *P. aeruginosa* in different types of meat.

Sort	Location	Meat type	No. of tested samples	No. of positive samples	Infection rate	Author
Retails	Cairo and Giza	Fresh minced meat resh sausage frozen beef burger	Fresh minced meat (*n* = 45), resh sausage (*n* = 30), frozen beef burger (*n* = 30)	Fresh minced meat (*n* = 32), fresh sausage (*n* = 13), frozen beef burger (*n* = 8)	Fresh minced meat: 71.11%, fresh sausage: 43.33% frozen beef burger: 26.67%	Amal et al. (2014)
	Mosul	Beef Mutton chicken	Beef (*n* = 50), mutton (*n* = 50), chicken (*n* = 50)	Beef (*n* = 23), mutton (*n* = 11), chicken (*n* = 19)	Beef: 46%, mutton: 22%, chicken: 38%	Tahr and Hasan (2022)
	Nigeria		100	3	3%	Alimi et al. (2022)
	Alborz province	Meat and meat product	370	29	7.83%	Rezaloo et al. (2022)
	Hong Kong, China	Pork and chicken	Chicken (*n* = 8), pork (*n* = 8)	Chicken (*n* = 5), Pork (*n* = 3)	Chicken:63%, pork: 38%	Wong et al. (2015)
	Deonar abattoir, Mumbai	Sheep/goat	116	11	10.2	Bhandare et al. (2007)
Abattoirs	Deonar abattoir, Mumbai	Sheep/goat	96	17	18%	Bhandare et al. (2007)
	Mazandaran and Golestan province, Iran	Raw meat and carcass surface swab	550	57	8.54%	Poursina et al. (2022)
	Egypt	Camel meat	200	45	22.5%	Elhariri et al. (2017)

### 4.4. Fruits and vegetables

Complex microbial communities also exist in a range of vegetables and fruits besides the foods described above (Li et al., 2020a,b,c,d), and endophytic and exterior parasitic *P. aeruginosa* has been normally isolated in fresh, raw vegetables and fruits, for example, cucumber, minitomatoes, tomatoes, onion, carrots, lettuces, spinaches, celeries, and different types of salads (Remington and Schimpff, 1981; Xu et al., 2019). *Pseudomonas* spp. (23%) was the dominant microbial agent found in both soft rot and whole lettuce samples (*n* = 100) in Nigeria (Erhirhie et al., 2020). Ezemba, C. C found that in salad and four other ready-to-eat samples on Uli campus, the probability of identifying *E.coli*, *Klebsiella pneumoniae*, and *P. aeruginosa* was 68.45%, 20.24%, and, 11.31%, respectively, (Ezemba et al., 2022). Raw and fresh salad vegetables act as a vector that transmits opportunistic *P. aeruginosa* to humans including immunocompetent people. Immunocompetent people, like cystic fibrosis patients, are particularly vulnerable to acute or chronic lung infections arising from *P. aeruginosa*. Therefore, it has been suggested that salads should not be consumed by high-risk patients (Remington and Schimpff, 1981; Table 3).

**Table 3:** Prevalence of *P. aeruginosa* in different types of vegetable.

Food type	Location	No. of tested samples	No. of positive samples	Infection rate	Author
Both soft rot and whole lettuce	Nigeria	100	23	23%	Erhirhie et al. (2020)
Salad	Uli campus			11.31%	Ezemba et al. (2022)
Raw vegetable	Spain	145	77	53.1%	Ruiz-Roldan et al. (2021)
Vegetables, fruits and sprouts	Mumbai, India	120	77	64.2%	Viswanathan and Kaur (2001)
Raw vegetable	Multan City, Pakistan	145	87	60%	Razzaq et al. (2014)
Lettuce	University restaurants of Spain	44	1	2.80%	Soriano et al. (2000)

## 5. The outbreak of *Pseudomonas aeruginosa* infection

*Pseudomonas aeruginosa* is increasingly known for its potential to cause outbreaks of diseases associated with public places such as hospitals and its resistance to a wide range of drugs. The results of the study showed that at least 10^6^ *P. aeruginosa* are required to establish a flora in the intestinal tract of a healthy adult. In patients with reduced colonization resistance due to antimicrobials, 1 gram of salad containing 10^3^ Gram-negative rods may be sufficient to cause sustained colonization of the intestine (Remington and Schimpff, 1981). *Pseudomonas aeruginosa* was examined in salads, cold meat, other cold food, and hot food, in public spaces including eight hospitals, two schools, and 11 canteens (Shooter et al., 1971). Most hospitals and canteens contain enough organisms to cause the swallowed organisms to build up in the bowel. Due to the contaminated hospital waste-water system, multidrug-resistant *P. aeruginosa* outbreaks, involving 89 patients, occurred in two English hospitals (File et al., 1995). Shooter et al. (1971) reported *P. aeruginosa* was detected in medicines and food, the organism is detected in the feces of patients after a period of time following the ingestion of food contaminated with the organism.

## 6. Antimicrobial resistant *Pseudomonas aeruginosa* has posed another concern for food safety

Continued overuse and misuse of antibiotics in food animals in order to treat disease or promote growth have triggered the emergence of drug-resistant superbugs and the increased mortality rates caused by bacterial infections pose a significant threat to human health (Xu et al., 2009; Kumar et al., 2020). Unlike another species of ESKAPE, *Staphylococcus aureus*, for which resistance primarily is mediated by an element in chromosome- SCC*mec* (Liu et al., 2016), resistance mechanisms in *P aeruginosa* has been commonly found to be associated with mobile elements, such as plasmids and integrons (Yu et al., 2016). Class 1 integrons have been commonly reported in *P. aeruginosa* since 1990s, then we had firstly reported class 2 integrons in *P. aeruginosa* in 2000s (Xu et al., 2009). Class 2 integrons were occasionally reported as we had also firstly reported class 1 integrons in *S. aureus* and 4 species of MRCNS (Xu et al., 2007, 2008, 2011a,b; Deng et al., 2015), class 1 and 2 integrons in *Enterococcus* (Xu et al., 2010), during similar period in the same medical setting. Since 2010s, we had observed more novel resistance genes on mega plasmids in *P. aeruginosa* (Liu et al., 2018a,b; Chen et al., 2019). Fluoroquinolone resistance is rapidly emerging in Gram-negative bacteria *Pseudomonas* spp., and the use of quinolones in food animal husbandry was found to be one of the main causes of the prevalence of fluoroquinolone-resistant *P. aeruginosa* (Gasink et al., 2006). In bovine meat (*n* = 230), fresh fish (*n* = 130), and smoked fish (*n* = 140), the prevalence of *P. aeruginosa* multidrug-resistance was 47.8%, 33.1%, and 20.0%, respectively (Vega-Mercado et al., 1995; Quintieri et al., 2019). *Pseudomonas aeruginosa* has a high level of drug resistance (Xie et al., 2017; Liu et al., 2018b), with the largest proportion of resistance to aztreonam being 36.4%, the following being ceftazidime, cefepime, and tobramycin. About one fifth of *P. aeruginosa* was resistant to beta-lactamases and about one tenth of *P. aeruginosa* was resistant to metallo-beta-lactamases and carbapenemases (Correa Rivas et al., 2015). The growing prevalence of resistance to carbapenem is a severe worldwide threat to public health, posing a risk to human and animal health. From minced meat in Egypt, one *P. aeruginosa* was isolated, which resists all antibiotics except colistin (Sadek et al., 2021). In Dhaka city, 100% of *P. aeruginosa* isolated from frozen meat and chicken nuggets showed resistance to ampicillin, penicillin, cefixime, and cefpodoxime, and three-tenths of isolates were not susceptible to cefotaxime (Bhuiya et al., 2018). The 19 *P. aeruginosa* isolates in milk samples (*n* = 125) obtained from dairy farms in Tirupati showed high resistance to ampicillin, penicillin, and oxacillin (100%; Swetha et al., 2017). In Jamaica, vegetable samples collected from supermarkets (55.6%) and groceries (72.3%) were extensively polluted with *P. aeruginosa*, which were resistant to ampicillin (100%), chloramphenicol (84%), trimethoprim (83%), and aztreonam (41%), with 35% of these isolates being insensitive to all four antibacterial compounds (Allydice-Francis and Brown, 2012).

## 7. Prevention and safety control of *Pseudomonas aeruginosa* in food

Preventing food spoilage and inhibiting the growth of pathogenic microorganisms is often achieved through the use of synthetic preservatives. However, the use of chemicals can be harmful to human health and can lead to the acquisition of resistance by microorganisms (Liu Z. et al., 2021). As a result of these concerns, there is an urgent need for scientists to find healthier, natural, effective, and environmentally friendly alternative antimicrobial agents. Various plant extracts belonging to plant secondary metabolites (PSMs) have therapeutic potential and are useful for the reduction of pathogens (Singh et al., 2003). Ethanolic extracts of *Punica granatum* peels and *Syzygium aromaticum* flowers exhibited inhibitory and germicidal activity and the MIC values for some of the more sensitive food borne *P. aeruginosa* range from 2.0 to 5.0 mg/mL (Mostafa et al., 2018). The essential oils extracted from basil, marjoram, oregano, rosemary, sage, grapefruit, citrus, lemon, *Thymus vulgaris*, *Mentha piperita, Eucalyptus globulus*, and *Lonicera japonica* could be used to inhibit *P. aeruginosa* virulence (Stojanović-Radić et al., 2016). Juglone from walnut husk, is highly effective, natural, non-hazardous, low residue and has the potential to inhibit *P. aeruginosa*. The test findings indicated that juglone at a concentration of 35 μg/mL was effective in inhibiting the colony formation of *P. aeruginosa* even at a concentration of around 10^7^ CFU/mL (Han et al., 2021).

The main reason why these PSMs control *P. aeruginosa* virulence is that the small molecules from a number of common plants and foods were obtained and selected for their QSI activity to try to combat the widespread opportunistic pathogen *P. aeruginosa*. Some bacteria modulate their phenotypes associated with pathogenicity *via* chemical signaling molecules, also termed quorum sensing (QS; Xu et al., 2021b; Liu et al., 2022a,b). The expression of genes related to QS in *P. aeruginosa* can be specifically inhibited by Iberin from Horseradish manufactured by a wide range of members of the *Brassicaceae* family (Jakobsen et al., 2012). In addition, biofilm formation inhibition and cell structure change can also effectively impede the development of *P. aeruginosa*. Because of the increased resistance of biofilm to disinfectants and bactericides, higher disinfectant concentrations and more contact time are needed to effectively inhibit bacterial activity. Carvacrol, available in the majority of antimicrobial essential oils, maybe a potential agent for QS inhibition because of its capacity to bind to proteins which are combined on cell membranes or involved in biofilm formation (Tapia-Rodriguez et al., 2017). Thymol is thought to be effective in inhibiting planktonic *P. aeruginosa* by disrupting cell integrity and increasing the permeability of the membrane, allowing the cell contents to escape (Liu T. et al., 2021).

Some other substances could also be utilized as healthy alternative preventive tools to control food spoilage due to microorganisms and to reduce the health risks posed by *P. aeruginosa*. Honey shows excellent antibacterial activity toward clinical bacterial samples of *P. aeruginosa* and *Escherichia coli* (Mandal et al., 2010). The growth and biofilm form of *P. aeruginosa* can also be inhibited by the papain hydrolysis product from the camel milk whey (Abdel-Hamid et al., 2020). Cell membrane disruption caused by Arg-Ser-Ser (RSS) handling results in leakage of intracellular contents from *P. aeruginosa* cells. Thus, RSS exhibits potential inhibition of *P. aeruginosa* (Liu et al., 2020).

## 8. Conclusion

As one of the most common foodborne pathogens, *P. aeruginosa* can cause severe acute and chronic life-threatening infections. Factors contributing to bacterial virulence include LPS, adhesins, agglutinins, and flagellates. *P. aeruginosa* often exists in various types of food, such as water, milk, meat, vegetable and fruit. Unfortunately, in the face of outbreaks of *P. aeruginosa* infections, overuse and misuse of antibiotic have made them resistant to antibiotics. The prevention of foodborne poisoning from *P. aeruginosa* is based on hygienic measures to avoid or reduce contamination of food by *P. aeruginosa*. Chemical preservatives can be used as appropriate to suppress the growth of *P. aeruginosa*. But as a typical biofilm former, *P. aeruginosa* attaches to a variety of surfaces or tissues, making it difficult to remove *P. aeruginosa* from food and medical instruments. Therefore, more research on this issue is urgently needed to be able to reduce the public health burden.

## Author contributions

XL writing—original draft and data curation. NG resources and conceptualization. TH methodology and supervision. FZ writing—review and editing. GP supervision and funding acquisition. All authors contributed to the article and approved the submitted version.

## Funding

This project was supported by grants from National Natural Science Foundation of China (82170056 and 81570045), Local Innovative and Research Teams Project of Guangdong Pearl River Talents Program (2017BT01S155), Natural Science Foundation of Guangdong Province (2021A1515011024, 2017A030313683, and 2014A030313486), Science and Technology Program of Guangzhou (202201020404 and 201510010226), National Key Research and Development Program of China (2018YFC1311600, 2018YFC1311604, 2016YFC1304100, and 2016YFC1304104).

## Conflict of interest

The authors declare that the research was conducted in the absence of any commercial or financial relationships that could be construed as a potential conflict of interest.

## Publisher’s note

All claims expressed in this article are solely those of the authors and do not necessarily represent those of their affiliated organizations, or those of the publisher, the editors and the reviewers. Any product that may be evaluated in this article, or claim that may be made by its manufacturer, is not guaranteed or endorsed by the publisher.
